# Developing a High School Mental Health Action Checklist in Thailand: insights on perception and communication

**DOI:** 10.3389/fpubh.2024.1437957

**Published:** 2024-10-09

**Authors:** Suwatsa Punneng, Sara Arphorn, Chatchai Thanachoksawang, Goontalee Bangkadanara, Chaiyanun Tangtong, Suchinda Jarupat Maruo, Niranyakarn Chantra, Densak Yogyorn, Tomohiro Ishimaru

**Affiliations:** ^1^Department of Occupational Health and Safety, Faculty of Public Health, Mahidol University, Bangkok, Thailand; ^2^Center of Excellence on Environmental Health and Toxicology (EHT), OPS, MHESI, Bangkok, Thailand; ^3^School of Health Science, Sukhothai Thammathirat Open University, Nonthaburi, Thailand; ^4^Department of Occupational Health and Safety, Faculty of Public Health and Environment, Huachiew Chalermprakiet University, Samut Prakan, Thailand; ^5^Department of Medical Humanities, School of Medicine, University of Occupational and Environmental Health, Japan, Kitakyushu, Japan

**Keywords:** action checklist, coping, high school students, mental health, new normal

## Abstract

**Background:**

This study aimed to develop a mental health action checklist for high school students that emphasized the importance of readiness, safety, and good hygiene at work to enhance their mental health and prepare them for the workforce.

**Methods:**

In total, 949 students from public high schools in Bangkok, Thailand voluntarily participated in this study. The 15-item High School Mental Health Action Checklist was developed based on a literature review and a pilot study. We used the index of item-objective congruence (IOC) to verify the content validity of the Checklist and exploratory factor analysis (EFA) to establish its construct validity. The tool’s internal consistency was estimated using Cronbach’s alpha coefficient.

**Results:**

The High School Mental Health Action Checklist had good content (IOC = 0.867) and construct validity. EFA revealed four factors that accounted for 46.5% of the variance. The overall reliability coefficient for the High School Mental Health Action Checklist was 0.788, and the reliability coefficients of the subdomains were 0.783–0.797.

**Conclusion:**

The Checklist will allow schools to develop an annual action plan for student mental health promotion activities, in accordance with public health guidelines. Our results indicate the High School Student Mental Health Action Checklist has good psychometric properties.

## 1 Introduction

Adolescence, a critical developmental stage between 10 and 24 years, is marked by heightened sensitivity to social situations and a strong need for peer interaction (1). The Coronavirus Disease 2019 (COVID-19) pandemic has sharply increased psychological distress among adolescents. This distress was partly attributable to reduced face-to-face social interactions and an increased reliance on digital communication. Importantly, this shift had lasting impacts on students’ mental health. In addition, economic challenges faced by families and the hybrid onsite/online learning model continue to influence students’ well-being in the post-pandemic environment (2, 3).

It is essential to consider theories of perception and communication, including behaviorist learning theories and cognitive approaches, to fully understand adolescents’ behaviors during this period. These theories can help explain how adolescents process information and develop mental skills. Understanding these aspects is crucial for addressing selective attention, which reflects how adolescents focus on specific stimuli in their complex social environments while ignoring others (4).

Existing mental health screening tools for secondary school students, though available (5–10), are not fully adequate under current circumstances for a thorough and consistent assessment of high school students’ mental health (11). Therefore, there is a need to develop a specialized tool that include problem-focused and emotion-focused coping strategies, considered effective in psychotherapy (12). It should allow students to express their individual mental health concerns through open-ended questions, facilitating genuine self-expression, which is vital during adolescence (13).

Action checklists, widely utilized as practical training tools in various workplaces, play a crucial role in helping participants identify areas for improvement. These checklists are designed to leverage the experiences and knowledge of participants, fostering the creation of viable solutions, lists of potential actions, or practical steps for progress (14). Participants are asked to learn from existing good examples identified in the discussion setting (15, 16).

Over the past two decades, the development of action checklists has been an ongoing process, with numerous studies affirming their effectiveness across different sectors (13–15, 17–19). The International Labour Organization (ILO) has contributed significantly to this field by creating various effective action checklists applicable in diverse areas (17). However, the application of mental health action checklists specifically tailored to certain age groups, such as high school-aged adolescents, remains relatively unexplored. Addressing the mental health needs of high school students is of paramount importance, considering their transitional phase into the workforce. In Thailand, most mental health assessments in educational settings are quantitative, and there is a lack of qualitative approaches that can offer deep insights and inform improvements. This means the development of a qualitative mental health action checklist is essential.

Therefore, this study aims to develop a mental health action checklist for high school students. Particularly, the objective is to provide high school students with a structured tool that enhances their understanding and awareness of mental health issues. This checklist is intended to serve as a practical training intervention, assisting in the improvement of mental health among adolescents preparing for the challenges of the workforce.

## 2 Methods

### 2.1 Sample size calculation

The Office of the Education Council reported that Thailand’s public schools accommodate 1,543,041 high school students aged 14–20 years (20). Of these, 116,430 high school students are enrolled in Bangkok (21). We focused on Bangkok because it is Thailand’s capital city and these students face high educational competition and have diverse economic, family, social, and intellectual backgrounds. The required sample size for this study was determined using the finite population correction for proportions. With a 99% confidence interval (CI), 5% margin of error, and maximum variability for the estimated proportion (*p* = 0.5), the target sample size was calculated as 662 students. We received voluntary registrations from interested students that exceeded this number meaning our sample included 949 high school students.

### 2.2 Participants

We conducted a cross-sectional study among high school students in Bangkok, Thailand. Participants were recruited using purposive sampling. After development, the High School Mental Health Action Checklist was distributed in schools in Bangkok (total population of 116,430 students aged 14–20 years). This study enrolled 949 high school students as volunteer participants from May to August 2022. Thailand implemented various restrictions in response to the COVID-19 pandemic that caused significant financial strain for many families. Students were previously accustomed to engaging in group activities with their families, schools, and communities, but experienced a sharp decline in these interactions following the implementation of these restrictions. The increased reliance on online activities created challenges in adapting to reduced face-to-face communication, which adversely affected students’ mental health (22). We first sought permission from four identified schools and asked them to promote participation in the research project and seek students’ consent to participate. Next, we emailed a link to the High School Mental Health Action Checklist to students that were interested in participating.

### 2.3 Developing the High School Mental Health Action Checklist

The High School Mental Health Action Checklist was developed based on a review of the literature relevant to mental health and stress screening and assessment by the Participatory Action-Oriented Training framework (16) and coping (1, 3), from which we created a list of semi-structured in-depth questions (7, 16, 23–26). In the next phase of development, we held comprehensive focus-group interviews with 11 experts and stakeholders, including mental health specialists, educational assessment and administration specialists, and teachers. These discussions were instrumental in drafting an initial version of the checklist, comprising 16 items specifically tailored for high school students’ mental health needs. To assess the reliability of this checklist (overall reliability coefficient of 0.661), we conducted a pilot test with 36 students. Concurrently, three experts evaluated the content validity of our draft, resulting in the refinement of the checklist to 15 items. Finally, the quality of these 15 items was rigorously assessed using the item-objective congruence (IOC) method (average 0.788).

### 2.4 Statistical analysis

In this study, we developed the Mental Health Action Checklist, focusing on how high school students cope with issues that impact their mental health. Descriptive statistics were used to examine participants’ characteristics, including sex, age, and grade. The IOC was used to verify the content validity of the questionnaire, and Cronbach’s alpha coefficient was used to estimate its reliability/internal consistency (theoretical range 0.00–1.00). The factor solution was determined based on the number of eigenvalues greater than 1. We conducted exploratory factor analysis (EFA) using 0.50 as the factor loading criterion to distinguish components of the High School Mental Health Action Checklist. All statistical analyses were performed with SPSS version 23.

## 3 Results

Participants’ (*N* = 949) demographic details are shown in Table 1. The majority of participating high school students were female (71.1%), 29.6% were aged 17 years (mean = 16.5 years, standard deviation [SD] = 1.1 years), and 41.2% were in Grade 10 (mean = 11.0, SD = 0.9).

**Table 1:** Participants’ demographic characteristics (*N* = 949).

	*n*	(%)	Mean (SD)
Sex			
Male	274	(28.9)	
Female	675	(71.1)	
Age (years)			16.5 (1.1)
14	3	(0.3)	
15	206	(21.7)	
16	280	(29.5)	
17	281	(29.6)	
18	171	(18.0)	
19	5	(0.5)	
20	3	(0.3)	
Grade			11.0 (0.9)
10	391	(41.2)	
11	212	(22.3)	
12	346	(36.5)	

SD, standard deviation.

The factor structure of the High School Mental Health Action Checklist is shown in Table 2. The IOC for the checklist was 0.867, with 15 items defined as requiring revision (IOC > 0.05). Reliability coefficients ranged from 0.783 to 0.797, with an overall score of 0.788. The EFA included data for all 949 participants, and used varimax rotation because the underlying constructs were conceptualized as independent of each other. The Kaiser–Meyer–Olkin measure of sampling adequacy was 0.749, and Bartlett’s test of sphericity was statistically significant, which suggested that the correlation matrix was suitable for factor analysis. Four meaningful constructs/factors emerged: (1) environmental impact (four items), (2) attachment and feeling inside (three items), (3) interaction and conflict (three items), and (4) disease/digital awareness (two items). These factors accounted for 46.51% of the cumulative variance. Three items did not load on any of the factors.

**Table 2:** Factor structure of the High School Mental Health Action Checklist (*N* = 949).

Action checklist	Factors				*h* ^2^	IOC	Reliability (Cronbach’s alpha)
	F1 (environmental impact)	F2 (attachment and feeling inside)	F3 (interaction and conflict)	F4 (disease/digital awareness)			
7	0.675				0.500	1	0.794
8	0.758				0.584	0.667	0.794
14	0.749				0.586	0.667	0.797
15	0.669				0.525	1	0.796
6		0.628			0.474	1	0.785
11		0.571			0.330	1	0.788
12		0.631			0.420	0.667	0.787
1			0.666		0.469	1	0.784
2			0.598		0.445	1	0.785
5			0.706		0.509	1	0.784
9				0.718	0.565	0.667	0.787
10				0.694	0.524	0.667	0.786
3					0.320	0.667	0.783
4					0.460	1	0.788
13					0.268	1	0.787
Eigenvalue	2.112	1.891	1.659	1.315			
% of variance	14.078	12.606	11.057	8.769			
Cumulative %	14.078	26.684	37.741	46.510			

IOC, item-objective congruence.

Table 3 presents the items included in the High School Mental Health Action Checklist. The Checklist asked students whether they were competent in relation to each item, and reliability coefficient testing resulted in 15-item summaries. Participants responded to this checklist without any empirical issues. All items were answered with “yes” or “no.” For higher-priority items, participants were also asked to write down their reasons or suggestions. A variety of responses were gathered regarding issues affecting participants’ mental health and their coping strategies. These insights were shared with the responsible teachers and used in further mental health promotion efforts at each school.

**Table 3:** Items in the High School Mental Health Action Checklist.

No	Items
1	Preparing a plan is for students to feel satisfied with their lives.
2	Preparing students to cope with disappointment.
3	Demonstrate the ability to cope with difficult problems.
4	Demonstrate the ability to help others when the opportunity arises and to make them feel happy or proud.
5	Demonstrate the ability to form loving and family ties that make them feel safe and comfortable with their families.
6	Demonstrate the ability to cope with conflict well when conflicts arise with those around them such as family members, friends, teachers, relatives, or anyone else.
7	Demonstrate the ability to live the new normal that will have an impact on their mental health.
8	Demonstrate the ability to reduce/relieve concerns about the excessive burden of online learning.
9	Demonstrate the ability to maintain social distancing in highly contagious epidemic situations.
10	Demonstrate the ability in Digital Intelligence Quotient to benefit without harming themselves or others.
11	Demonstrate the ability to interact more online, such as attending classes, attending school events, buying and selling products and meeting new acquaintances and new people online.
12	Demonstrate the ability in health literacy (access, understand, ask questions, make decisions, practice health).
13	Demonstrate the ability to make student life expectations (study, work, family, daily life, etc.).
14	Demonstrate the ability to manage environments that affect their expectations and happiness, such as environmental, health, cultural, social or political situations, etc.
15	Demonstrate the ability to relieve/care for fatigue, muscle pain of the body from studying or working, such as tiredness/burning eyes, blurred vision, headache, neck, back, shoulder pain, etc.

There is a guideline for answering all questions with two options (yes and no), and specifying the priority.

## 4 Discussion

The High School Mental Health Action Checklist was developed using the basic concepts of the ILO action checklist and demonstrated good content validity, construct validity, and reliability. Therefore, it is possible for individual students, schools, or those involved in remedial action to use the tool as a guide and method to promote mental health in schools (17).

Our study aligns with previous research indicating that EFA of similar tools typically reveals multiple factors. For instance, a recent relevant study identified five factors across 30 of 35 items, accounting for 42% of the variance. These factors included skills in seeking mental health information (27), consistent with another study focusing on the mediating role of COVID-19 coping strategies in anxiety and general health relationships (28). Our EFA distinguished three COVID-19-specific dimensions: perceived mental health impact (nine items: Cronbach’s *α* = 0.85), fear of COVID-19 (six items: *α* = 0.73), and positive coping (five items: *α* = 0.61). One item concerning the pandemic’s financial impact was excluded due to low factor loadings (29). This approach mirrors the structure of another action checklist encompassing 30 items across six technical areas, ranging from collaboration planning to workplace supervision (17). The High School Mental Health Action Checklist was designed to remain relevant and adaptable beyond the COVID-19 pandemic. Given that online and hybrid learning environments continue to be part of the educational landscape, this checklist will help address the ongoing mental health challenges associated with such settings. Moreover, the checklist is intended to be a dynamic tool, and items can be continually refined and updated on the basis of practical feedback following implementation in schools. This will ensure the long-term applicability and effectiveness of the instrument.

The High School Mental Health Action Checklist is expected to facilitate communication between students and teachers regarding mental health issues. Past studies have shown that action checklists can transform workplace environments, suggesting practical actions for environmental screening (18). They also facilitate rapid workplace-level discussions for effective screening (19). In our trials, some cases led to counseling or hospital treatment. For example, students who showed signs of depression or suicidal ideation received immediate support through counseling, with hospital referrals in severe cases. Later, as schools recognized the importance of mental health, they began planning training, assigning school counselors, and meeting with parents. It is noteworthy that the High School Mental Health Action Checklist that was developed in this study is used in these schools for regular mental health monitoring. Further research is necessary to confirm our checklist’s effectiveness in enhancing the school environment to improve high school students’ mental health and investigate the long-term impact on students’ decisions and well-being.

The present study had some limitations. First, this article focused on describing the development of the High School Mental Health Action Checklist, which was found to be easy to use, convenient, and accessible for student participants. It offers a questionnaire that can be used to screen students’ ability to cope with problems when encountering situations that affect their mental health. The results of the Checklist assessment were used to improve and promote the mental health of participating students. However, we cannot show that effect in this paper because the evaluation of the outcomes related to improving and promoting mental health using our Checklist has not yet been finalized. Second, this study was conducted in high schools located in and around Bangkok, Thailand. Therefore, caution should be exercised in generalizing the findings to other regions. Further research is necessary to evaluate the applicability of the High School Mental Health Action Checklist in different contexts, such as in other countries and different age groups, to ensure its effectiveness and adaptability across various settings. Third, the survey was conducted during the period of transition from online to on-site classes following the lifting of COVID-19 lockdown restrictions, and the timing of the survey could have influenced the results obtained. Therefore, further surveys are recommended to examine the universal reliability and validity of the checklist.

## 5 Conclusion

This study focused on the development of the High School Mental Health Action Checklist. This 15-item tool assists in screening coping mechanisms used to address students’ mental health challenges. Our findings showed that the tool had good validity and reliability. Students independently responded to the questionnaire, and the insights derived from their responses offered invaluable information for educators. Schools can therefore use these findings as a foundation or guideline for devising initiatives to promote students’ mental well-being. Regular and frequent use of the checklist to screen students’ mental health and plan interventions will allow schools to ensure that timely and effective interventions are offered. Encouraging a dynamic exchange of knowledge between teachers and students is crucial for enhancing the mental health of high school students.

## Data Availability

The original contributions presented in the study are included in the article/supplementary material, further inquiries can be directed to the corresponding author.
